# Management of acromegaly beyond primary surgery: efficacy and safety of repeat surgery and radiotherapy

**DOI:** 10.1007/s00701-025-06640-2

**Published:** 2025-08-18

**Authors:** Shahriar Atai, Markus Wiedmann, Daniel Dahlberg, Jens Bollerslev, Ansgar Heck

**Affiliations:** 1 Department of Specialised Endocrinology, Oslo University Hospital, Oslo, Norway; 2 Faculty of Medicine, University of Oslo, Oslo, Norway; 3 Department of Neurosurgery, Oslo University Hospital, Oslo, Norway

**Keywords:** Acromegaly, Somatotroph adenoma, Transsphenoidal surgery, Pituitary deficiency, Radiotherapy, Repeat surgery

## Abstract

**Introduction and purpose:**

Re-intervention, either transsphenoidal surgery or radiotherapy, is suggested in patients who are not in remission after primary surgery for acromegaly; however, the evidence is weak. We aimed to assess the remission rate after re-intervention, and complications compared to a comparison group who had undergone primary interventions only.

**Methods:**

Patients diagnosed with acromegaly between 2005–2021 at Oslo University Hospital were screened for inclusion. The study cohort included patients with two or more interventions. The comparison group included patients not in remission after primary surgery.

**Results:**

Of 223 patients with acromegaly, 42 underwent re-interventions (study cohort). At diagnosis, median age was 38 (IQR 29–48) years and 41 patients (98%) had a macroadenoma. The comparison group consisted of 49 patients, median age 54 (IQR 44–60) years and 37 (76%) had a macroadenoma at diagnosis.

Re-interventions in the study cohort consisted of surgery, radiotherapy and a combination of these (22, 12 and eight patients). After re-interventions, 22 patients (52%) were in remission and 12 (29%) had reduced disease activity. Seven patients (17%) acquired new hormone deficiencies, two of them corticotroph deficiency. One patient in remission developed spinal fluid leakage and meningitis. There was no significant difference in complications after surgery between the study cohort and the comparison group.

**Conclusion:**

Re-interventions were safe and resulted in remission or substantial improvement in most patients. Re-intervention should be considered for patients who would otherwise require lifelong medical treatment.

## Introduction

Acromegaly is nearly always caused by a pituitary somatotroph adenoma, most often a macroadenoma [25]. If left untreated it is associated with severe morbidity and increased mortality, therefore focused treatment is essential and is often handled in tertiary referral centers by multiple disciplinary teams (MDT’s) [11]. The recommended primary therapy for most patients is transsphenoidal surgery, the only potentially curative treatment modality [20]. However, remission rates after primary surgery are only around 50%, with large adenomas and parasellar invasiveness being some of the contributing factors [5, 10, 15]. Most patients not in remission after primary surgery are recommended medical therapy [14, 20]. Modern medical therapy is efficacious, however demanding, costly, with burdensome adverse effects and is potentially life-long [8, 13]. Other treatment options are secondary interventions with repeat surgery or radiotherapy (re-intervention) [14, 20]. Re-interventions are only a suggestion in the Endocrine Society guidelines due to the scarcity of clinical evidence for its efficacy and safety [20]. For meaningful guidance of multimodal treatment including re-intervention, and shared decision making, studies on clinical outcome are needed.

Radiotherapy is often reserved to patients with uncontrolled disease after surgery and medical therapy. According to guidelines, it may take many years to develop full effect that moreover may be limited in some patients and the complication rates are high [14, 20]. Nonetheless, if re-intervention is efficacious, it may lead to complete remission and save patients from lifelong medical treatment.

The intention of re-intervention may depend on certain factors. In patients with small intrasellar residual adenomas and secretory activity, the intention is typically medication free remission without pituitary deficiency. On the other hand, patients with large invasive adenomas, debulking procedures due to biochemical activity, growth and/or compression of adjacent structures including the visual apparatus may be necessary.

The aim of our study was to assess the efficacy of re-intervention, and to compare safety outcomes with primary surgical interventions in a comparison group.

## Material and methods

### Patients

Patients diagnosed with acromegaly between January 1 st 2005 and end of June 2021 were identified from the pituitary registry at Oslo University Hospital. From these, patients with two or more interventions (primary surgery followed by repeat surgery and/or radiotherapy) were included in the study cohort. These patients required medical therapy due to persistent disease activity after primary surgery. The decision for re-intervention was based on the assessment of the clinical physician, shared decision making and MDT meetings. The intentions of re-intervention were recorded prospectively as part of clinical workup. We included a comparison group not in remission after primary surgery for acromegaly to describe the consequences of individual clinical selection for re-intervention versus continued medical therapy at group level. We compared the frequency of complications between the groups. As in the study cohort, the patients in the comparison group required medical therapy due to persistent GH excess after primary surgery. Data until end of June 2024 were included and all relevant visits including the last visit before this date were assessed. Patients not operated, in remission after primary surgery or with failure to follow-up were excluded. Patients were followed according to the observational protocol of the pituitary registry and routine clinical practice.

### Outcome and Biomarkers

The outcome was assessed at the last visit before end of June 2024 and was classified as follows:

- Remission was defined as no need for medication for acromegaly and IGF-1 within 1.3 × the upper limit of normal (ULN) [14],
- Reduced disease activity was defined as reduced dosage of medication due to improved IGF-1 concentrations,
- No effect = none of the two above

### Complications

Registered complications were pituitary deficiency, cerebrospinal fluid (CSF) leakage, meningitis, vascular injury, cranial nerve damage and venous thromboembolism in relation to the interventions.

In our pituitary registry pituitary deficiency is defined as one or more deficiencies that do not resolve spontaneously and have a typical biochemical pattern requiring hormone replacement therapy in accordance with Endocrine Society guidelines on hypopituitarism [12]. We separated the incidence of deficiencies depending on whether they appeared before or after an intervention, and after which intervention they occurred.

CSF leakage was defined as a leakage that required postoperative surgical intervention.

Meningitis was registered as a postoperative complication in patients with a clinical infection and findings of elevated leukocytes in CSF.

Vascular injury was defined as a bleeding in need of surgical intervention in the sellar and/or parasellar region.

Cranial nerve injury was defined as clinical signs of it including vision impairment, diplopia and sensory impairment due to injury of the trigeminus nerve.

Venous thromboembolism was deemed as a complication if it occurred within three months after pituitary surgery.

### MRI

MR imaging was performed routinely or as clinically indicated. The images were assessed at diagnosis as micro- (< 10 mm) or macroadenoma (≥ 10 mm), invasiveness according to the Knosp-Steiner criteria [21], volume (ellipsoid formula: craniocaudal x transverse x anteroposterior dimensions × 0.5) and signal intensity in T2 weighted images. They were classified as hyper-, iso or hypointense compared to the pituitary gland, alternatively to gray matter if the pituitary gland was not visible [18].

### Statistics

Median and interquartile range (IQR) were used for age, follow-up time, assessment of biomarkers and adenoma size in milliliters (ml). Pearson Chi Square and Fisher Exact Test were used for the differences between the two groups regarding adenoma size (macro- or microadenomas), invasiveness, MRI T2 intensity, medical therapy prior to primary surgery and their difference in safety. Fisher exact test was employed to test the association between invasiveness and outcome in the study cohort. The Wilcoxon-Rank sum test was conducted to assess differences in age, follow-up time, biomarkers, adenoma size in ml and duration of medical therapy prior to primary intervention between the two groups.

## Results

**Fig. 1:**
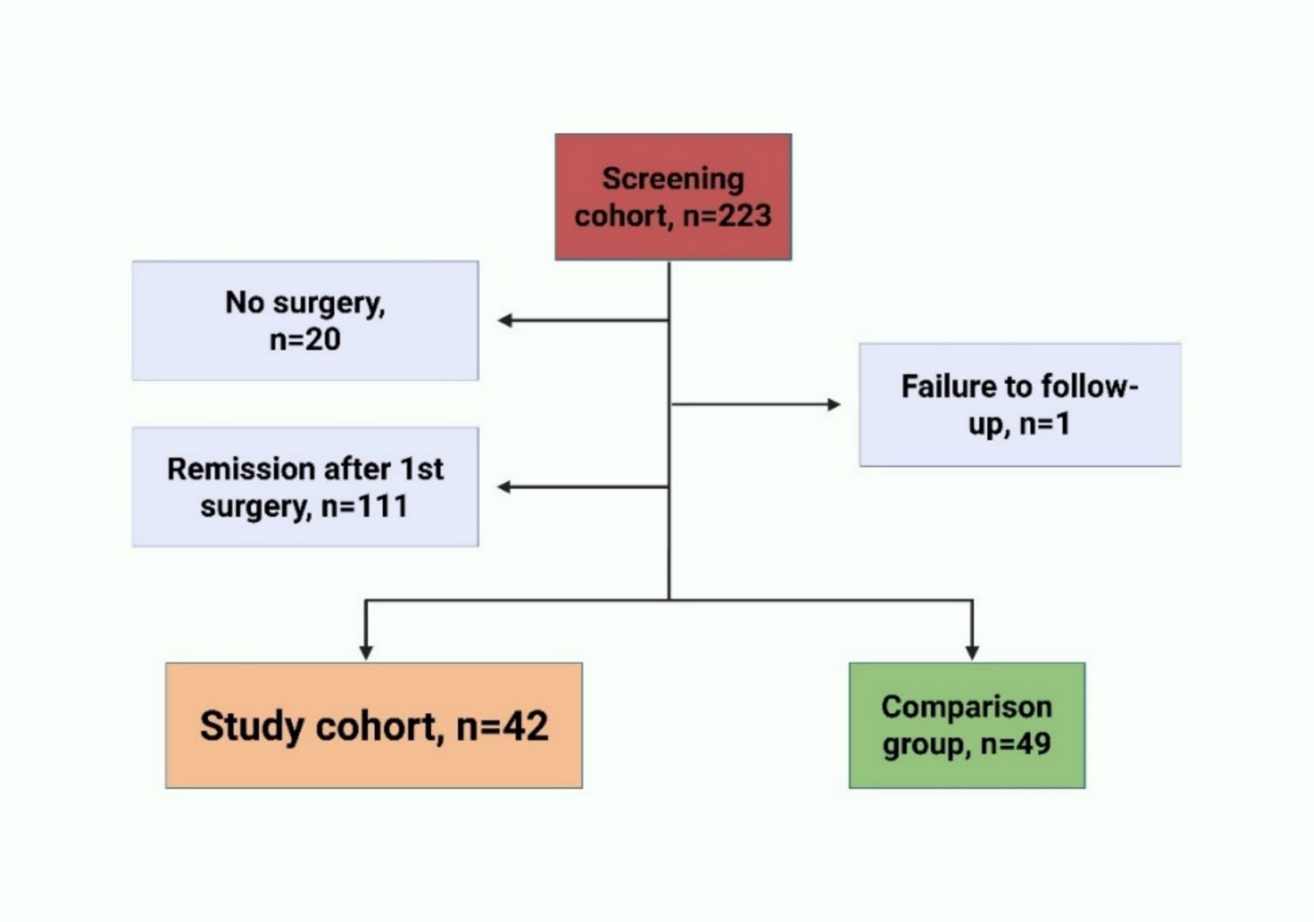
Study cohort and the comparison group. The figure shows the screening cohort of patients diagnosed with acromegaly between January 1 2005 and 30 June 2021. The study cohort were patients who had undergone two or more interventions. The comparison group were patients not in remission after primary surgery. Patients in remission after primary surgery were excluded. Twenty patients who had not undergone surgery due to several reasons such as surgeon or patient preference were excluded. One patient was lost to follow-up. Created in Biorender

Of the 223 patients diagnosed with acromegaly between 2005 and 2021, 42 had undergone secondary interventions and were included in the study cohort. Forty-nine patients with primary interventions only and not in remission were included in the comparison group (Fig. 1). The characteristics of the study cohort and the comparison group at baseline are presented in Table 1. The patients in the study cohort were younger with larger, more hyperintense and more invasive adenomas. They had lower GH concentrations; however, there was no difference in IGF-1/upper limit of normal (ULN) between the groups.

**Table 1:** Characteristics of the study cohort and the comparison group at baseline

	**Frequency (%)/median (IQR)**		**P-value**
**Characteristics**	**Study cohort**	**Comparison group**	
**Gender Female/Male**	21/21	24/25	0.9203
**Age**	37.5 (29–47.5)	51 (44–60)	0.0002
**Follow-up years**	10 (7–13.75)	10 (7–15)	0.8293
**IGF-1/ULN**	2.7 (1.9–3.3)	2.9 (2.1–3.7)	0.2465
**GH µg/L**	16 (7–23)	7.7 (4.9–14.3)	0.0312
**Prolactin IU/L**	379 (168–688)	377 (237–1041)	0.1641
**Size adenoma**			
Macroadenoma	41 (98%)	37 (76%)	0.005
Microadenoma	1 (2%)	11 (22%)	
n/a		1 (2%)	
ml	2.7 (1.6–5.1)	0.9 (0.3–3.3)	0.0003
**Knosp**			0.084
0–2	28 (66.7%)	38 (78%)	
3–4	14 (33.3%)	8 (16%)	
n/a		3 (6%)	
**MRI T2 intensity**			0.024
Hypointense	16 (38%)	30 (61%)	
Other	21 (50%)	14(29%)	
n/a	5 (12%)	5 (10%)	
**Pre-treatment**			0.348
Yes	23 (55%)	22 (45%)	
No	19 (45%)	27 (55%)	

Follow-up time: years from baseline to the last visit. ULN: upper limit of normal. Knosp: the invasiveness of the adenoma after the Knosp-Steiner criteria. Pre-treatment if the patients received medical therapy prior to primary surgery: In the study cohort first generation somatostatin analogues (SSA; n = 19), SSA and pegvisomant (n = 2), dopamine agonists (DA; n = 1), SSA and DA (n = 1). In the comparison group SSA (n = 18), SSA and DA (n = 3), SSA and pegvisomant (n = 1). Median duration of pre-treatment: 12 (IQR 8–19) and 16 (IQR 7–21) months in cohort and comparison group respectively with no significant difference in duration (p = 0.61)

**Fig. 2:**
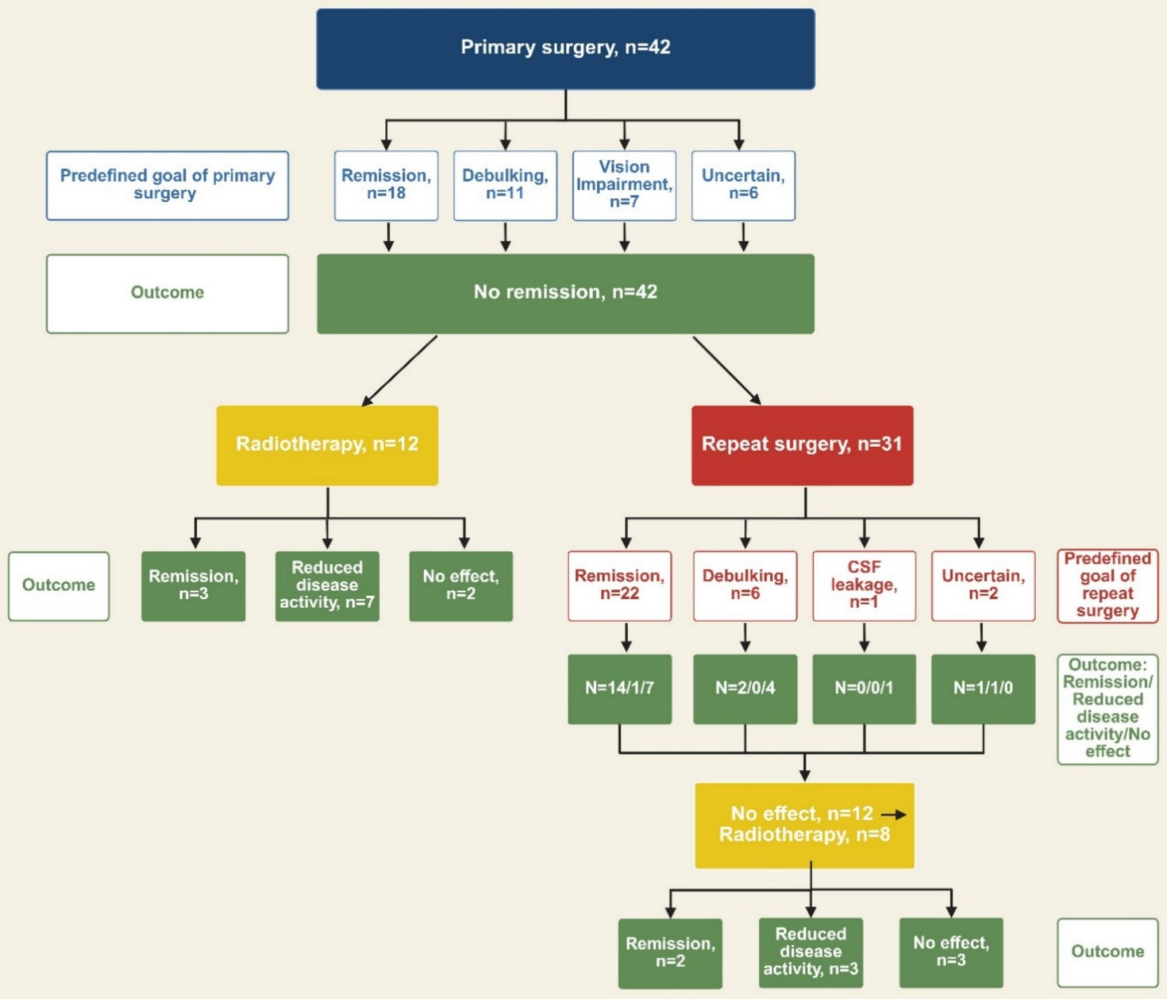
Study cohort: Interventions, their predefined goals and outcomes. In total, repeat surgery was performed 31 times on 30 patients, one patient had two repeat surgeries. Four of the surgeries where craniotomies, one as primary surgery and two craniotomies were performed on the same patient. One patient underwent repeat surgery due to CSF leakage; however, the residual adenoma was also removed during the same procedure. Created in Biorender

The predefined goals of surgery for the study cohort are presented in Fig. 2. In the comparison group, 48 patients underwent primary surgery. In 33 of these, the predefined goal was biochemical remission. Additional goals were relief from adenoma mass effects including optic apparatus affection in three, debulking in one and uncertain in eleven patients. One patient underwent primary radiotherapy.

In total, 51 re-interventions were performed in the 42 patients after primary surgery. The re-interventions consisted of surgery (n = 31), either transsphenoidal (n = 28) or transcranial surgery (n = 2) or a combination of the two techniques (n = 1).

Intraoperative Doppler was routinely used during all surgical procedures, and all patients underwent endoscopic resection apart from the two who underwent transcranial surgery without simultaneous transsphenoidal surgery. Resection of the medial wall of the cavernous sinus was performed in four patients in the cohort, three during repeat surgery, all of whom were in remission, and one during primary surgery. The procedure was not performed in any patients in the comparison group.

Intraoperative MRI was performed in ten patients in the cohort, seven during primary surgery and three during repeat surgery, which did not lead to further surgery. It was performed in seven patients in the comparison group of whom one was further operated during the same procedure.

Radiotherapy modalities included photon therapy, either fractionated therapy (n = 7, 1.8 Gy × 28–30) or stereotactic radiosurgery (n = 6, 20–25 Gy × 1), proton beam therapy (n = 4, 1.8 Gy × 29–30) or gamma knife radiosurgery (n = 3, 20–32 Gy × 1, in one patient the dosage was not available).

The median follow-up time in patients after repeat surgery without subsequent radiotherapy was 3.3 (IQR 1.3–5.8) years. The median follow-up time after radiotherapy was 6.7 (IQR 3.9–11.2) years. If performed, radiotherapy was always the last modality.

### Efficacy

**Fig. 3:**
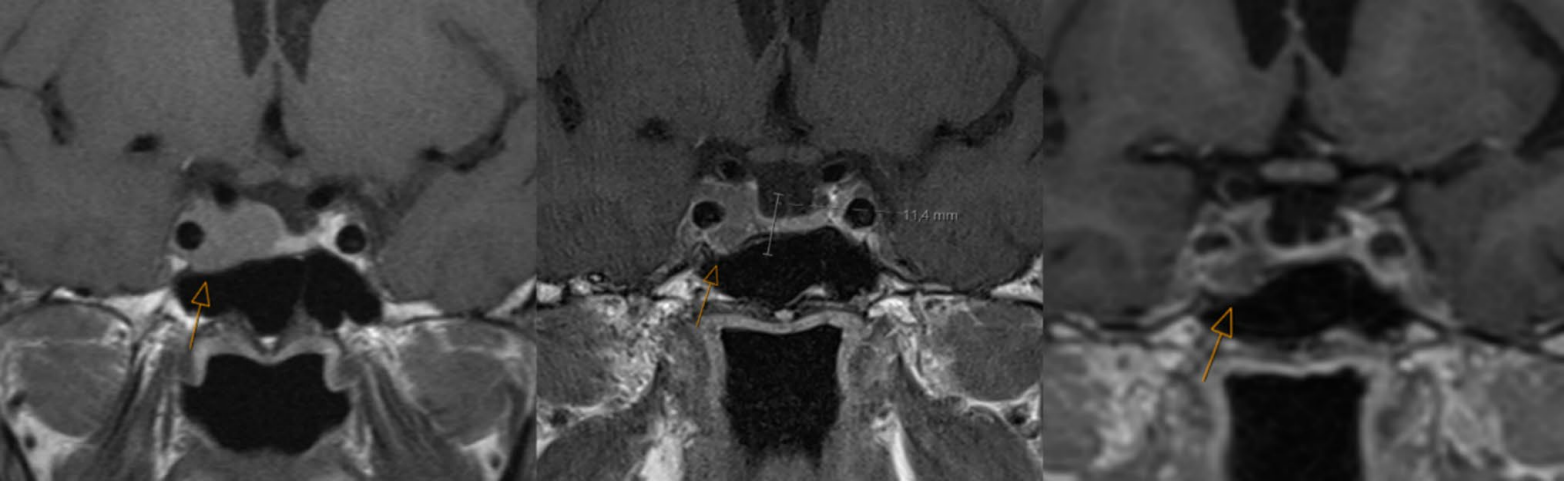
T1 weighted MR images enhanced with gadolinium. A patient who at baseline in 2010 (image to the left) had an invasive macroadenoma (arrow) and underwent primary surgery in 2012. He was not in remission and a residual adenoma was visualized (image in the middle, arrow). He had high IGF-1 concentrations and experienced side effects on medical therapy. Through a multi-disciplinary team meeting, he was referred to radiotherapy since surgery was inappropriate due to invasiveness. He was in remission after radiotherapy which he received in 2016. The image to the right from 2023 shows a finding (arrow) that is merely enhanced by gadolinium

**Fig. 4:**
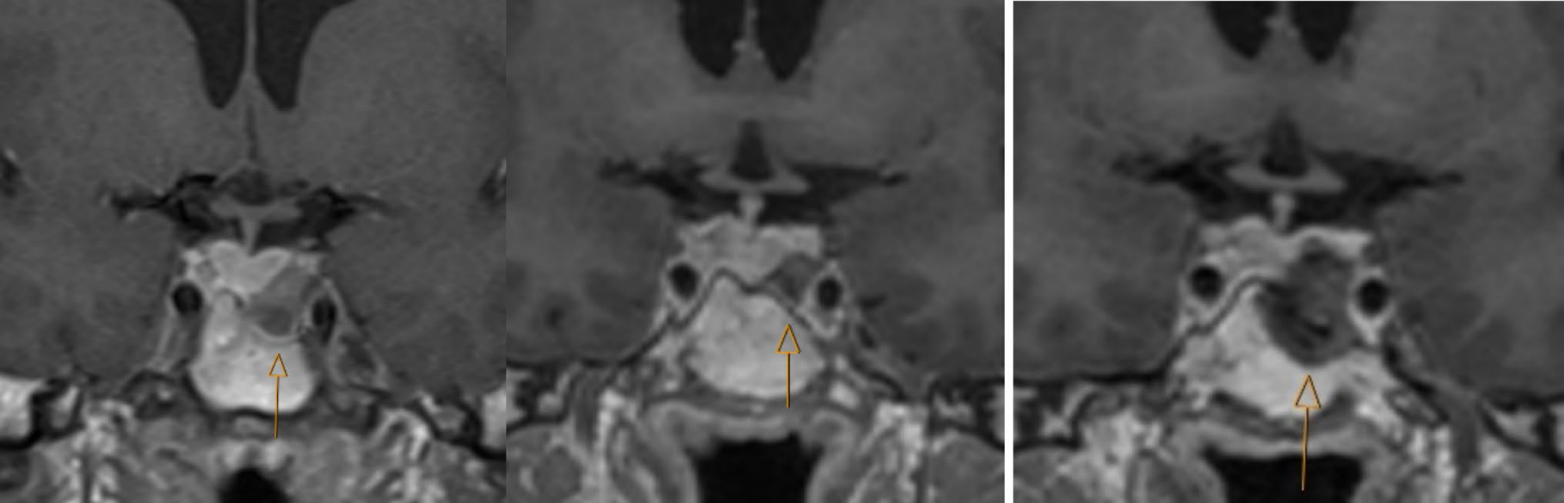
T1 weighted images enhanced with gadolinium. A patient with a macroadenoma at baseline (image to left, arrow) underwent primary surgery in 2015. He was not in remission and medical therapy was indicated. A residual adenoma was visualized (image in the middle, arrow) and through an MDT meeting and shared decision making, the patient underwent repeat surgery in 2023. The MR image to the right is one day after repeat surgery showing postoperative changes (arrow) with no visible residual adenoma. He was in remission after surgery, including at the last visit in 2024

After re-interventions, 22 patients (52%) were in remission, 12 (29%) could reduce the dose of medication because of reduced disease activity. Eight patients (19%) had no effect of re-intervention. Five of 20 patients who received radiotherapy were in remission and ten could reduce the dosage of medical therapy (Table 2). Among the five patients who did not have effect following radiotherapy, two had a short follow-up time after the treatment (0.7 and 1.5 years). In 22 patients, remission was the predefined goal of re-intervention. Of these, 14 (64%) were in remission after repeat surgery (Fig. 2). Figures 3 and 4 show MR images of two patients with invasive adenomas who achieved remission after re-intervention. Notably, patients in remission in our study had a normalized IGF-1 within or equal to the ULN.

**Table 2:** Summary of number of patients, sequence of re-interventions and outcomes

Re-intervention(s)	Frequency (%)	Remission	Reduced disease activity	No effect
Radiotherapy	12 (29%)	3	7	2
Surgery	22 (52%)	17	2	3
Surgery + radiotherapy	7 (17%)	1	3	3
2 surgeries + radiotherapy	1 (2%)	1	0	0
**Total n**	**42**	**22**	**12**	**8**

Primary surgeries for all patients (n = 42) are not included in the table. If radiotherapy was given, this was the last modality

One patient was in remission after craniotomy via eyebrow incision and supraorbital access, one was in remission after craniotomy and additional radiotherapy. One patient with the combined surgical techniques had active disease at the last visit, however the patient underwent radiotherapy in October 2023.

### Predictors of remission

Adenomas with a Knosp grade 0–2 at diagnosis had a significantly higher remission rate after re-intervention compared to invasive adenomas grade 3–4 (p = 0.049, Table 3). Of the four patients in remission with invasive adenomas, three had a Knosp 3 grade and one had a Knosp 4 grade adenoma. Three of these received radiotherapy, and one (Knosp grade 3, microadenoma) was in remission after repeat surgery. Of the ten patients with an invasive adenoma not in remission, four had a Knosp grade 3 and six had a Knosp grade 4 adenoma.

**Table 3:** Adenoma invasiveness at baseline and outcome after re-intervention, p = 0.049

Study cohort	Remission	No remisison	Total n
Knosp 0–2	18	10	28
Knosp 3–4	4	10	14
**Total n**	22	20	42

We found no significant correlation between gender, age, prolactin concentrations, IGF-1/ULN, medical treatment before primary surgery and MRI T2 intensity at baseline and remission.

### Safety

Seven patients acquired new pituitary deficiencies in one or more axes, including two cases of corticotroph deficiency following re-intervention. Repeat surgery resulted in new deficiencies in five patients including one corticotroph deficiency. After radiotherapy, three patients acquired hormone deficiencies, one was corticotroph deficiency. In one patient, repeat surgery caused hormone deficiency, and subsequent radiotherapy led to new deficiencies including corticotroph deficiency. Accordingly, eight new pituitary deficiencies were recorded after re-intervention. Table 4 summarizes the different hormone deficiencies acquired after re-intervention and their outcomes.

**Table 4:** New hormone deficiencies after re-intervention

Hormone deficiency, n = 7	Outcome
Thyrotroph (radiotherapy)	Remission
Somatotroph, Gonadotroph (surgery)	Reduced
Thyrotroph (surgery)	No effect
Gonadotroph, Thyrotroph (surgery)	Remission
Thyrotroph (radiotherapy)	Reduced
Gonadotroph, Thyrotroph (surgery), Corticotroph, Somatotroph (radiotherapy)	Remission
Corticotroph, Gonadotroph (surgery)	Reduced

Reduced: reduced disease activity

There were no significant differences in new pituitary and corticotroph deficiencies after re-intervention in the study cohort compared to deficiencies after primary interventions in the comparison group. Eight new deficiencies, of these two corticotroph, were registered after re-intervention compared to five new deficiencies, of these two corticotroph, in the comparison group (P = 0.42 and p = 1, respectively).

There were no significant differences in pituitary and corticotroph deficiencies when adding the safety of primary surgeries in both groups compared to re-interventions in the study cohort (p = 0.82 and 0.49, respectively).

One patient acquired cerebrospinal fluid leakage and meningitis after repeat surgery; nonetheless, he recovered, and his acromegaly was in remission. There were no episodes of vascular injury, cranial nerve injury or venous thromboembolism after repeat surgery (Table 5).

**Table 5:** Complications in the study cohort and the comparison group

Complications	Cohort, n = 42 (%)	Comparison, n = 49 (%)	P-value
New pituitary failure after 1 st surgery	8 (19%)	5 (11%)	0.23
-Corticotroph failure	5 (12%)	2 (5%)	0.24
-Vasopressin deficiency (permanent)	1 (2%)	1 (2%)	1
New pituitary failure after 2nd intervention or later	7 (17%)	n/a	
-Corticotroph failure	2 (5%)	n/a	
-Vasopressin deficiency (permanent)	0 (0%)	n/a	
CSF leakage after 1 st surgery	1 (2%)	1 (2%)	1
CSF leakage and meningitis after 1 st surgery	1 (2%)	1 (2%)	1
CSF leakage and meningitis after 2nd surgery	1 (2%)	n/a	
Vascular injury	0 (0%)	0 (0%)	1
Venous thromobembolism	0 (0%)	1 (2%)	1
Cranial nerve injury after intervention	0 (0%)	0 (0%)	1
Deceased	0 (0%)	0 (0%)	1

Cohort: Study cohort. Comparison: comparison group. One patient acquired hormone deficiency after the first surgery, the same patients acquired new deficiency after re-intervention. Thus, the total number of patients acquiring hormone deficiencies in the cohort group after all interventions were 14. One patient acquired hormone deficiency after repeat surgery, later the patient acquired new hormone deficiency after radiotherapy. N/a: not available since the control group had not undergone second interventions. CSF: cerebrospinal fluid. Two patients had hormone deficiency, both gonadotroph, in the study cohort before any intervention; one resolved after primary surgery. Ten patients had hormone deficiency before primary intervention in the control group; five of them resolved after primary intervention

## Discussion

The present study shows that re-intervention leads to remission or reduced need of medical therapy in the majority selected patients with acromegaly. The risk of complications after re-interventions were low despite large and invasive adenomas.

So far, only a few studies have described the results of repeat surgery in patients with acromegaly. Our data are in accordance with a correspondent Japanese study showing that repeat transsphenoidal surgery on 53 patients had a similar remission rate (58.5%) with a low incidence of complications [27]. However, the cohort was based on patients operated between 1987–2006, before multimodal therapy was generally accessible. A small study found the same magnitude of remission (57%) and confirmed the low incidence of complications [26]. A systematic review on repeated pituitary surgery in general [19] and a single center study regarding remission and complications of transsphenoidal surgery for acromegaly [5] showed similar results. The latter study focused on primary surgery as well as re-intervention with a total remission rate of 58%. We have summarized the surgical outcomes from different publications in Table 6. A systematic review concluded that patients with recurrent acromegaly might benefit more from medical therapy and radiotherapy compared to surgery [24]. However, the study concluded that the evidence was low and future studies on this patient group warranted. Several studies have shown that radiotherapy seems effective and safe [3, 7, 16, 23]. Over time, radiotherapy techniques have developed with more precise radiation delivery [16]. Likewise, surgical techniques have been refined and improved, such as endoscopy rather than microsurgery, four-hand procedures, intraoperative MRI and usage of a Doppler probe to avoid vascular injury, which has improved safety and also efficacy however to a lesser extent [6].

**Table 6:** Outcomes after repeat surgery for acromegaly

Publication	Remission rates	Complication rates
Kurosaki M et al. 2003 [22]	13/22 (59%)	None*
Espinosa de Los Monteros AL et al. 2009 [9]	5/38 (9%)**	NA
Yamada S et al. 2010 [27]	31/53 (59%)	Hypopituitarism, n = 1 (1.9%). CSF leakage with meningitis, n = 1(1.9%). Pituitary abscess n = 1 (1.9%)
Alahmadi H et al. 2012 [2]	4/9 (44%)	CSF leakage and meningitis, n = 1 (2.5%) Vascular injury, n = 1 (2.5%), Acute coronary syndrome, n = 1 (2.5%)***
Wilson TJ et al. 2013 [26]	8/14 (57%)	Aseptic meningitis, n = 2 (14%)
Almeida JP 2017 [4]	7/11 (64%)	None
Bengtsson OF et al. 2023 [5]	60/104 (58%)****	CSF leakage 8%, meningitis 4%, sepsis 2%, neurological deficits < 1%, bleeding < 1%, DVT/PE < 1%, surgical mortality < 1%*****

Different criteria for remission may have been applied. *No pituitary deficiencies were seen, and major complications were not seen, however these were not specified. **In additional n = 30 (57%) and n = 38 (72%) IGF-1 and GH decreased, respectively. ***Complications rates were for the whole series of patients (n = 39) including other pituitary adenomas. ****Remission rate for primary and repeat surgery for acromegaly in total. *****In total 578 patients with pituitary adenomas and their complications after transsphenoidal surgery

Our data show that the risk for complications by re-intervention was comparable to primary interventions. Most patients experiencing new pituitary deficiencies did achieve either remission or reduced disease activity. These safety findings can be reassuring in the process of shared-decision-making and help to balance potential benefits of re-intervention against risks. However, the selection process towards repeat surgery included a neurosurgical and anesthesiologic risk assessment.

The strengths of this study are the large cohorts and long and complete follow-up by a standardized observational protocol. We stratified our results according to pre-defined intention of re-intervention, which to our knowledge has not been fully applied in previous studies. The retrospective non-randomized single center nature of the study may influence its external validity as might the clinical selection of patients for both cohorts. However, the study describes daily clinical workup and care of consecutive patients with acromegaly and real-life data over time in a detailed fashion. It is a limitation that the two presented cohorts are not statistically comparable. The younger patients in the study cohort had larger T2-hyperintense adenomas. Such patients may require secondary interventions more frequently due to resistance to medical therapy compared to patients in the comparison group with smaller T2-hypointense adenomas [1, 17]. In addition, older patients may not be candidates for secondary interventions due to higher surgical risks and frailty. Despite the mentioned differences between the groups, safety outcomes were similar. Besides pituitary deficiency, other potential long-term complications after radiotherapy were not assessed.

In conclusion, we consider re-intervention a viable alternative to lifelong medical treatment for many patients as it results in remission in the majority of cases and poses a low risk of complications. For patients requiring debulking procedures, re-interventions are a safe and integral component of modern multimodal treatment, promoting long-term control of acromegaly. Our study results may provide a stronger foundation for recommendations in MDT’s and shared decision-making.

## Data Availability

The data is collected from our registry and the medical records of the participants, and the analyses are stored as files at a sensitive data localization. The data cannot be openly shared due to the privacy of the study participants as well as the data protection policy of the institution. Upon reasonable request, the data may be shared by the corresponding authors.
